# Discriminatory performance of total calcium for ionized hypocalcemia and factors affecting ionized calcium in Assaf ewes during the pre- and post-lambing periods

**DOI:** 10.1093/jvimsj/aalag202

**Published:** 2026-09-07

**Authors:** Kenan Ç Tümer, Sébastien Buczinski, Serkan Bozaci, Ömer Deniz, Tarik Şafak, Berrak I Deniz, Abdullah Şimşek, Thomas Wittek

**Affiliations:** Department of Internal Medicine, Faculty of Veterinary Medicine, Kastamonu University, 37150 Kastamonu, Turkey; Département des Sciences Cliniques, Faculté de Médecine Vétérinaire, Université de Montréal, St-Hyacinthe, J2S 2M2, Canada; Department of Internal Medicine, Faculty of Veterinary Medicine, Kastamonu University, 37150 Kastamonu, Turkey; Department of Internal Medicine, Faculty of Veterinary Medicine, Kastamonu University, 37150 Kastamonu, Turkey; Department of Obstetrics and Gynecology, Faculty of Veterinary Medicine, Kastamonu University, 37150 Kastamonu, Turkey; Department of Obstetrics and Gynecology, Faculty of Veterinary Medicine, Kastamonu University, 37150 Kastamonu, Turkey; Ihsangazi Vocational School, Kastamonu University, 37150 Kastamonu, Turkey; Clinical Centre for Ruminant and Camelid Medicine, Clinical Department for Farm Animals and Food System Science, University of Veterinary Medicine Vienna, 1210 Vienna, Austria

**Keywords:** calcium metabolism, diagnostic discordance, postpartum, prepartum, sheep

## Abstract

**Background:**

Total calcium concentration (tCa) poorly reflects ionized hypocalcemia across species, and multiple factors influence ionized calcium concentration (iCa^2+^). These relationships have not been evaluated in Assaf ewes.

**Hypothesis/Objectives:**

Assess the diagnostic accuracy of tCa for detecting hypocalcemia and identify factors associated with iCa^2+^ during pre- and post-lambing periods.

**Animals:**

A total of 105 clinically healthy multiparous Assaf ewes from a commercial flock**.**

**Methods:**

Prospective observational study. Venous blood samples were collected during pre- and post-lambing periods. Blood gas-electrolytes (pH, pCO₂, pO₂, HCO₃^−^, TCO₂, Na^+^, K^+^, iCa^2+^) and biochemical analyses (total protein, albumin, tCa, magnesium, phosphorus, β-hydroxybutyrate [BHB], glucose, alkaline phosphatase) were performed. Discriminatory performance of tCa was evaluated using area under the receiver operating characteristic curve (AUC) analysis. Variables associated with iCa^2+^ were assessed using multiple linear regression.

**Results:**

During the pre-lambing period, tCa showed moderate diagnostic performance (AUC, 0.73; 95% CI, 0.57-0.87; sensitivity, 67%; specificity, 80%) at a cutoff of ≤ 2.42 mmol/L, whereas performance was poor for the post-lambing period (AUC, 0.59; 95% CI, 0.47-0.70; sensitivity, 97%; specificity, 31%) at ≤ 2.77 mmol/L. In multiple regression, tCa, BHB, pCO₂, and pO₂ were associated with iCa^2+^ during pre-lambing (adjusted R^2^ = 0.27, *P* < .001), whereas tCa, BHB, phosphorus, albumin, and Na^+^ were associated with the post-lambing period (adjusted R^2^ = 0.25, *P* < .001).

**Conclusions and clinical importance:**

iCa^2+^ varied by production stage and was influenced by several physiologic variables. Direct measurement of iCa^2+^ should be prioritized to improve the accuracy of hypocalcemia assessment in Assaf ewes.

## Introduction

Late gestation and early lactation impose substantial and rapidly shifting demands on calcium metabolism in ewes. The requirements for fetal skeletal mineralization followed by the onset of milk production place ewes at risk of negative calcium balance even in the absence of overt clinical disease.^1^ Hypocalcemia during these stages has been linked to impaired uterine contractility, dystocia, fetal hypoxia, metabolic dysregulation, and increased lamb and ewe mortality.^2–5^

In practice, calcium status is routinely evaluated using serum or plasma tCa.^6^ This approach assumes that tCa reliably reflects biologically active calcium. However, tCa comprises protein-bound, complexed, and ionized fractions, whereas only the ionized fraction exerts biological effects.^7^^,^^8^ Evidence from cattle indicates that the relationship between tCa and iCa^2+^ is not constant but varies according to health state, production stage, acid–base balance, plasma protein concentrations, and mineral status.^9–11^ Additionally, tCa demonstrated limited discriminative ability to detect ionized hypocalcemia in sick adult cattle and cattle in the early postpartum period.^10^^,^^11^

In ewes, hypocalcemia has been traditionally evaluated as a disorder of late gestation.^1^ However, calcium efflux associated with the onset of lactation may equal or exceed that observed during pregnancy, indicating that calcium homeostasis remains under substantial challenge beyond parturition.^1^^,^^12^^,^^13^ A previous study reported serial measurements of tCa and iCa^2+^ from 10 days before parturition to 10 days postpartum.^13^ Although tCa remained relatively stable across time points, iCa^2+^ increased progressively toward parturition, peaked at 6 h postpartum, and subsequently decreased, reaching its lowest concentration by 10 days postpartum. That study did not specifically aim to evaluate the relationship between calcium fractions. However, the divergent trajectories of tCa and iCa^2+^ suggest potential stage-dependent dissociation between them. Despite these observations, neither the stage-specific association between tCa and iCa^2+^ nor the influence of acid–base status, mineral balance, plasma protein concentrations, and metabolic indicators on iCa^2+^ has been systematically evaluated in ewes. We therefore investigated the relationship between plasma tCa and blood iCa^2+^ during the final weeks of gestation and the postpartum period, and evaluated the biochemical determinants of iCa^2+^ during these stages. We hypothesized that (1) the association between tCa and iCa^2+^ would differ between prepartum and postpartum periods, and that tCa would not consistently reflect iCa^2+^ status across these stages; and (2) changes in acid–base status, plasma protein concentrations, mineral balance, and metabolic indicators would significantly influence iCa^2+^.

## Materials and methods

The details for synchronization, feeding, blood sampling, laboratory, and statistical analysis are presented in Appendix 1.

### Animals

Our study was designed as a prospective observational study and performed on 105 late-pregnancy multiparous Assaf ewes from a single commercial flock. All experimental procedures were conducted after approval by the Kastamonu University Ethics Committee for Animal Experiments (23/05/2025, Decision No: 39).

### Blood sampling and laboratory analyses

Blood samples from 105 ewes were collected during pre- and post-lambing periods. In each sampling period, blood was collected from the jugular vein of each ewe. Samples were obtained under anaerobic conditions into syringes containing dried heparin for blood gas-electrolyte analysis, K₃EDTA tubes for packed cell volume (PCV) determination, and lithium heparin tubes for biochemical analyses. Blood gas-electrolyte analysis was performed using a portable blood analyzer (i-STAT 1, Abbott Point of Care, Abbott Laboratories, Chicago, IL) with single-use CG8+ cartridges (Abbott Point of Care, Abbott Laboratories, Chicago, IL), previously validated for use in sheep.^14^^,^^15^ The PCV of the collected blood samples was determined using microhematocrit centrifugation method at 13 687 × *g* for 5 min as previously described.^16^ Biochemical analyses including alkaline phosphatase, tCa, total protein, albumin, glucose, BHB, phosphorus, and magnesium were performed using an automated chemistry analyzer (Respons 910, Diasys Diagnostic System GmBH, Holzheim, Germany) with commercially available reagent kits from the same manufacturer (Diasys Diagnostic System GmBH).

### Statistical analysis

Statistical analyses were performed using RStudio (Version 4.5.2, R Foundation for Statistical Computing, Vienna, Austria). The normality of the continuous variables was evaluated using the Shapiro–Wilk test. The differences in continuous variables between the pre- and post-lambing periods were assessed using the Wilcoxon rank-sum test. Optimal cut-off values of tCa for the identification of hypocalcemia were determined separately for the pre- and post-lambing periods using *cutpointr* package in R.^17^ Hypocalcemia in both periods was defined as a blood iCa^2+^ < 1.1 mmol/L.^18^ Receiver operating characteristic (ROC) curves were constructed for both periods, and the area under the receiver operating characteristics curve (AUC) was used to quantify discriminatory performance. Internal validation and variability evaluation of the estimated tCa cut-off values were performed using a bootstrap resampling. The associations between blood iCa^2+^ and both measured and calculated variables including blood pH, pCO₂, pO₂, Na^+^, K^+^, TCO₂, HCO₃^−^, AP, tCa, phosphorus, magnesium, total protein, albumin, glucose, and BHB were evaluated separately using multivariable linear regression analysis for the pre- and post-lambing periods.

### Results

The median interval between blood sampling and lambing was 10 days (interquartile range [IQR], 6-18 days), whereas the median time of sampling after lambing was 18 days (IQR, 10-22 days). In the study population of 105 multiparous ewes, litter size distribution was as follows: single lambing in 27 ewes (25.7%), twin lambing in 72 ewes (68.6%), and triplet lambing in 6 ewes (5.7%).

### Blood-gas and plasma biochemical analysis results

The medians and IQR (Q1-Q3) for blood gas and plasma biochemical variables, along with the results of the Wilcoxon rank-sum test, are presented in Table 1. Compared with the post-lambing period, the pre-lambing period was characterized by lower pH, pCO₂, pO₂, HCO₃^−^, TCO₂, tCa, magnesium, and phosphorus concentrations, but higher Na^+^ and iCa^2+^ concentrations. Intra-assay coefficient of variation (CV) values for blood pH, pCO₂, pO₂, TCO₂, HCO₃^−^, Na^+^, K^+^, and iCa^2+^ were 1.2%, 2.48%, 2.36%, 1.24%, 1.42%, 1.32%, 0.94%, and 1.04%, respectively. The intra-assay and inter-assay CVs were, respectively, 1.39% and 2.76% for tCa, 2.96% and 4.71% for magnesium, 1.49% and 2.08% for phosphorus, 3.62% and 4.48% for AP, 2.14% and 2.72% for BHB 2.38% and 3.04% for glucose, 1.42% and 2.42% for total protein, and 1.67% and 1.98% for albumin.

**Table 1:** Blood gas-electrolyte and selected plasma biochemical variables (median, Q1-Q3) and Wilcoxon rank-sum test results during the pre- and post-lambing periods (*n* = 105).

Variable	Pre-lambing	Post-lambing	*P*-value
**pH**	7.45 (7.43-7.47)	7.48 (7.45-7.50)	<.001
**pCO_2_ (mmHg)**	36.5 (35.1-38.0)	37.9 (35.9-40.4)	.01
**pO_2_ (mmHg)**	33.0 (30.0-36.0)	35.0 (32.0-38.0)	.004
**HCO_3_ (mmol/L)**	25.40 (24.30-26.20)	28.00 (26.30-29.80)	<.001
**TCO_2_ (mmol/L)**	26.00 (25.00-27.00)	29.00 (27.00-31.00)	<.001
**Na^+^ (mmol/L)**	146.00 (145.00-147.00)	143.00 (141.00-144.00)	<.001
**K^+^ (mmol/L)**	4.90 (4.70-5.20)	4.90 (4.60-5.20)	.3
**iCa^+2^ (mmol/L)**	1.17 (1.13-1.21)	1.13 (1.06-1.18)	.002
**tCa (mmol/L)**	2.48 (2.38-2.53)	2.62 (2.52-2.74)	<.001
**iCa^+2^/tCa**	0.48 (0.45-0.50)	0.43 (0.39-0.45)	<.001
**Magnesium (mmol/L)**	0.98 (0.74-1.09)	1.06 (1.00-1.13)	<.001
**Phosphorus (mmol/L)**	1.34 (1.05-1.53)	1.92 (1.75-2.15)	<.001
**β-OHB (mmol/L)**	0.54 (0.43-0.70)	0.50 (0.42-0.59)	.04
**Glucose (mmol/L)**	3.80 (3.51-4.16)	3.39 (3.11-3.62)	<.001
**Total Protein (g/dL)**	6.64 (6.39-7.15)	7.32 (6.88-7.58)	<.001
**Albumin (g/dL)**	3.30 (3.20-3.44)	3.40 (3.25-3.52)	.01
**Globulin (g/dL)**	3.40 (3.10-3.70)	3.90 (3.50-4.20)	<.001
**AP (U/L)**	112 (86-143)	102 (62-150)	.2
**PCV (%)**	30 (26-34)	30 (27-35)	.4

### Discriminatory performance of total calcium for ionized hypocalcemia

**Figure 1:**
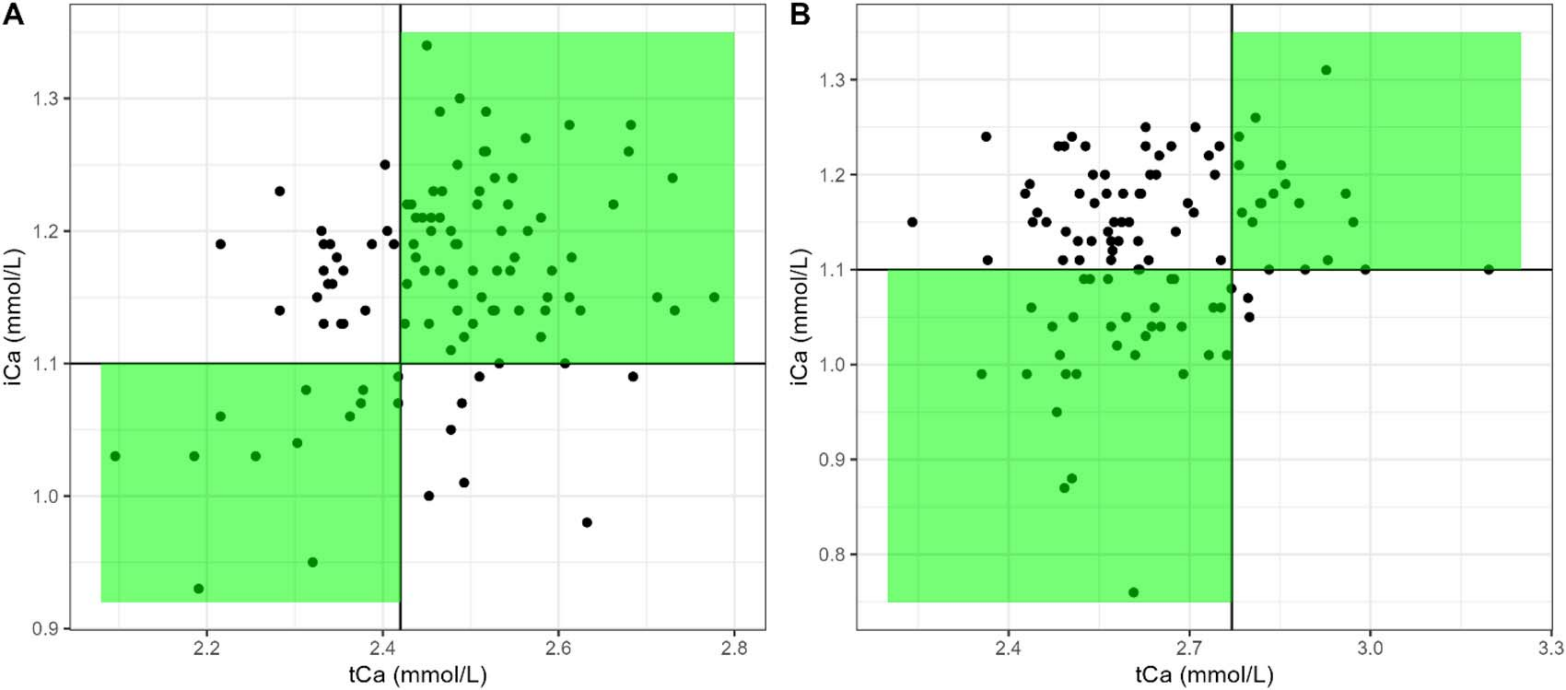
Relationship between plasma tCa and blood iCa concentrations in Assaf ewes during pre-lambing (A) and post-lambing (B) periods. Each point represents an individual animal. The vertical and horizontal black lines indicate the cut-off values for tCa and iCa, respectively (iCa < 1.1 mmol/L; tCa cut-off ≤ 2.42 for pre-lambing period and ≤ 2.80 mmol/L for post-lambing period). Green shaded areas represent concordant classifications (true positive and true negative), whereas non-shaded areas indicate discordant classifications (false positive and false negative) between tCa and iCa.

**Figure 2:**
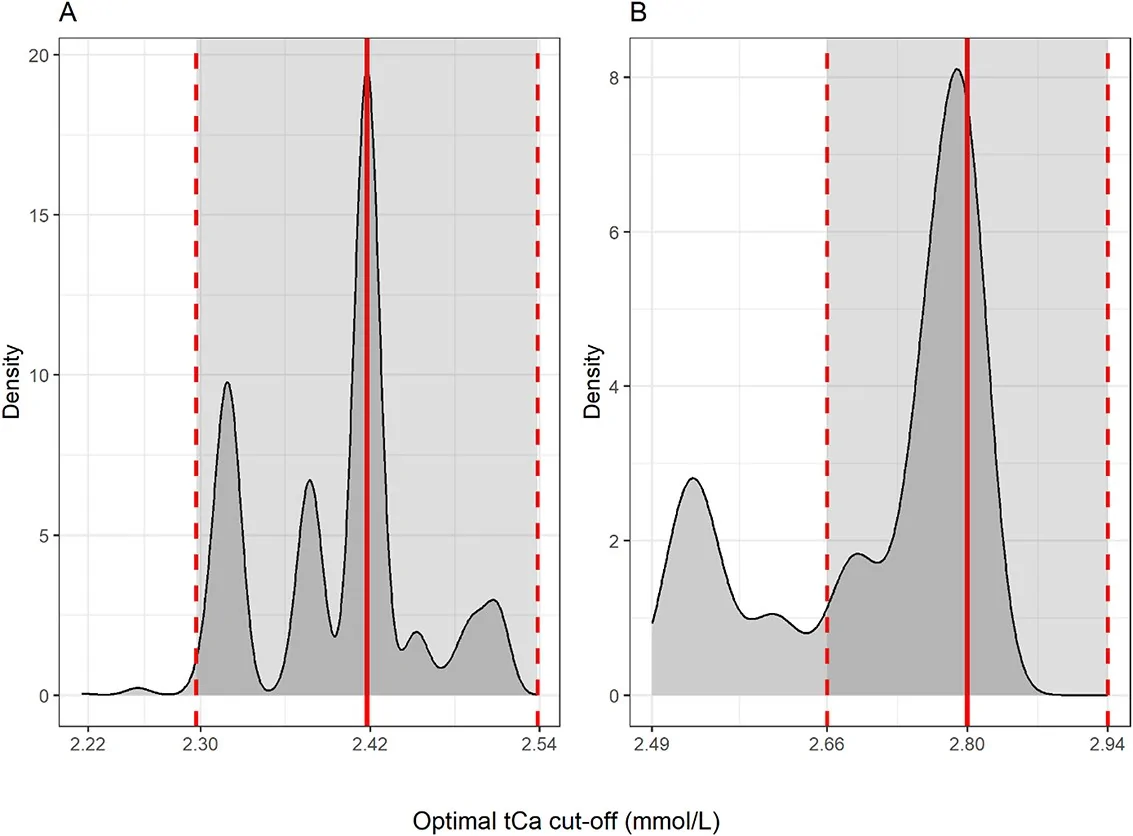
Distribution of bootstrap-derived optimal total calcium (tCa) cut-off values for the identification of hypocalcemia in the pre-lambing (A) and post-lambing (B) datasets. The solid red vertical line represents the optimal tCa cut-off estimated from the original dataset. The dashed red vertical lines indicate the predefined region of practical equivalence (ROPE) interval. The density curves illustrate the distribution of cut-off values obtained from bootstrap resampling.

Based on blood iCa concentrations (<1.1 mmol/L), 20 of 105 ewes (19.0%) were classified as hypocalcemic and 85 (81.0%) as normocalcemic during the pre-lambing period. In the post-lambing period, 34 of 105 ewes (32.4%) were identified as hypocalcemic, whereas 71 (67.6%) were normocalcemic (Figure 1). The optimal tCa cut-off concentration for detecting hypocalcemia during the pre-lambing period was ≤2.42 mmol/L. At this threshold, the AUC was moderate (0.73), with a sensitivity of 0.65, specificity of 0.76, and overall accuracy of 0.74. Bootstrap resampling of the pre-lambing dataset yielded a median optimal cut-off concentration of ≤2.42 mmol/L (95% CI, 2.30-2.51 mmol/L). Notably, 99.4% of the bootstrap-derived cut-off concentrations fell within the region of practical equivalence (ROPE) interval (±5% of the original optimal cut-off; Figure 2). The performance metrics of the optimal cut-off and the bootstrap-derived optimal cut-offs from pre-lambing data, evaluated in both in-bag and out-of-bag samples, are presented in Table 2.

**Table 2:** Diagnostic performance of optimal and bootstrap-derived cut-offs for total calcium during pre- and post-lambing periods.

Period	Data Source	Cut-off (mmol/L)	AUC	Se (95% CI)	Sp (95% CI)	Acc (95% CI)
Pre-lambing	Original Data	2.42	0.73	0.65	0.76	0.74
	In-bag	2.42 (2.30-2.51)	0.73 (0.57-0.87)	0.67 (0.35-0.96)	0.80 (0.41-0.99)	0.78 (0.51-0.91)
	Out-of-bag		0.73 (0.52-0.92)	0.50 (0.09-1.00)	0.78 (0.33-1.00)	0.74 (0.43-0.89)
Post-lambing	Original Data	2.80	0.59	1.00	0.23	0.48
	In-bag	2.77 (2.51-2.80)	0.59 (0.47-0.70)	0.97 (0.39-1.00)	0.31 (0.17-0.86)	0.53 (0.40-0.74)
	Out-of-bag		0.58 (0.43-0.74)	0.87 (0.14-1.00)	0.27 (0.12-0.84)	0.47 (0.34-0.64)

Abbreviations: Acc = accuracy; AUC = area under the curve; Se = sensitivity; Sp = specificity.

In the post-lambing period, the optimal tCa cut-off for discriminating hypocalcemia was ≤2.80 mmol/L. At this threshold, the AUC was 0.59, with a sensitivity of 1.00, a specificity of 0.23, and an overall accuracy of 0.48. Bootstrap resampling of the post-lambing dataset yielded a median optimal cut-off tCa of ≤2.77 mmol/L (95% CI, 2.51-2.80 mmol/L). Overall, 73.8% of the bootstrap-derived cut-off concentrations fell within the ROPE interval. The performance metrics of the optimal cut-off and the bootstrap-derived optimal cut-offs from post-lambing data, evaluated in both in-bag and out-of-bag samples, are summarized in Table 2.

### Association between blood iCa^2+^ and blood gas as well as selected biochemical variables during pre- and post-lambing periods

The results of the univariable analysis are presented in Table S1. Based on the multivariable modeling, the final model for the pre-lambing dataset included tCa, albumin, BHB, PCO_2_, and PO_2_. For the post-lambing dataset, the final model included total protein, albumin, BHB, tCa, Na^+^, and phosphorus. Model statistics for the linear models fitted to the pre- and post-lambing datasets are presented in Tables 3 and 4. Results for the nonlinear models also are provided in Tables S2 and S3.

**Table 3:** Linear multivariable regression models (with and without albumin) of the association between blood iCa^2+^ and selected biochemical variables in the pre-lambing dataset (*n* = 105).

	iCa ^2+^		iCa ^2+^			
Predictors	Estimates	95% CI	*P*-value	Estimates	95% CI	*P*-value
**(Intercept)**	0.22	−0.13 to 0.58	.21	0.34	0.02-0.67	**.04**
**Albumin**	0.05	−0.01 to 0.11	.1			
**BHB**	−0.02	−0.04 to -0.00	**.03**	−0.03	−0.05 to −0.00	**.02**
**tCa**	0.18	0.07-0.30	**.002**	0.21	0.10-0.32	**<.001**
**PCO_2_**	0.00	0.00-0.00	**<.001**	0.00	0.00-0.00	**<.001**
**PO_2_**	0.01	0.00-0.01	**<.001**	0.01	0.00-0.01	**<.001**
**Observations**	105		105			
**R^2^/R^2^ adjusted** ***P*-value**	0.302/0.267 <.001		0.283/0.254 <.001			

The bold numbers indicate statistical differences.

**Table 4:** Linear multivariable analysis of the association between blood iCa^2+^ and selected biochemical variables in the post-lambing dataset (*n* = 105).

	iCa ^2+^		
Predictors	Estimates	95% CI	*P*-value
**(Intercept)**	−0.67	−1.85 to 0.51	.26
**Albumin**	−0.07	−0.13 to −0.01	**.03**
**BHB**	−0.13	−0.22 to −0.04	**.01**
**tCa**	0.15	0.04-0.25	**.01**
**Na^+^**	0.01	0.01-0.02	**.001**
**Phosphorus**	−0.08	−0.11 to −0.04	**<.001**
**Observations**	105		
**R^2^/R^2^ adjusted** ***P*-value**	0.283/0.247 <.001		

The bold numbers indicate statistical differences.

**Figure 3:**
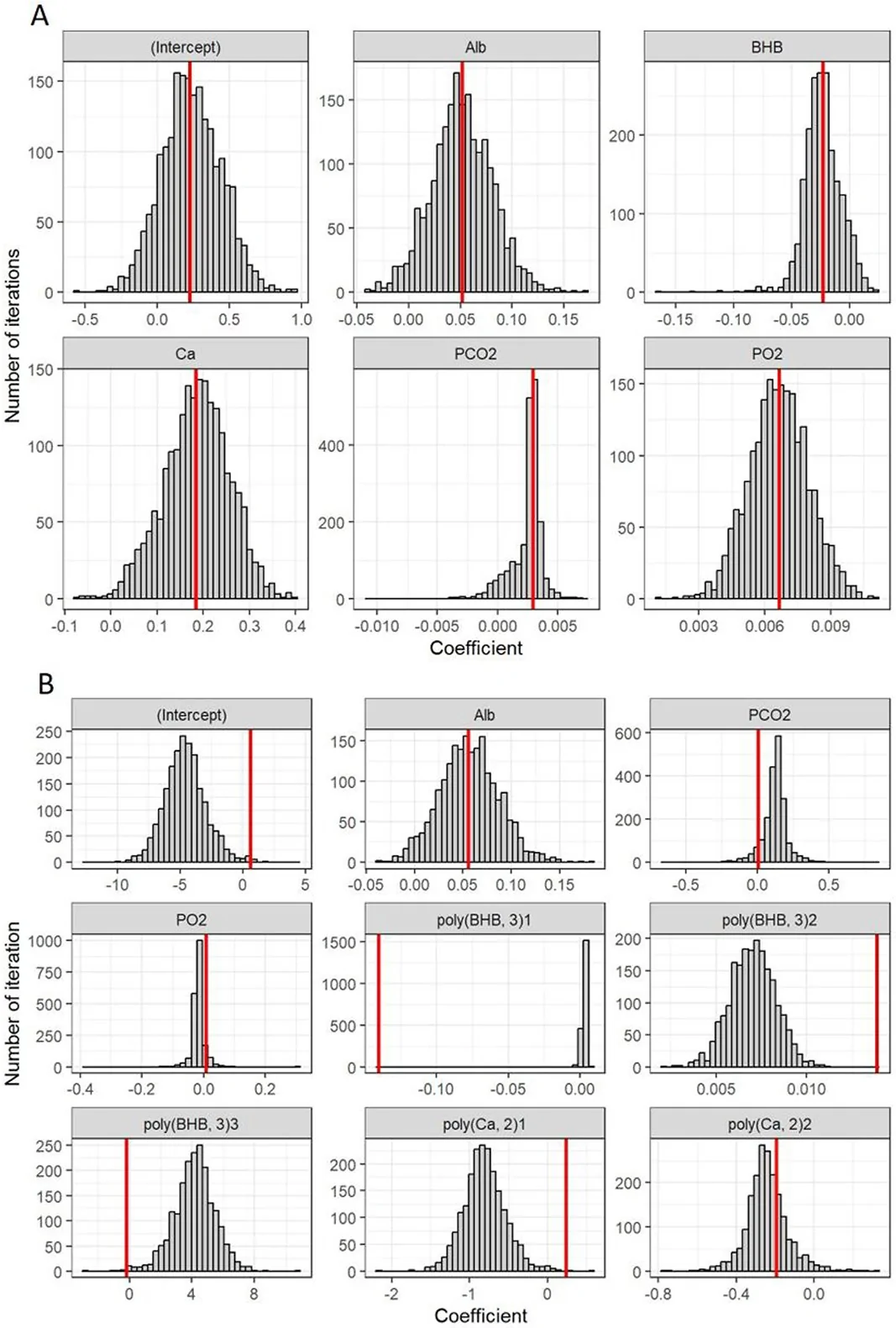
Histograms show the bootstrap distributions of regression coefficients obtained from 2000 resamples for the pre-lambing data, including linear (A) and nonlinear (B) models. Each panel corresponds to the intercept and predictor variables retained in the final models. The red vertical line represents the coefficient estimate from the original dataset. The degree of agreement between the original estimate and the bootstrap distributions indicates the stability and robustness of each predictor.

**Figure 4:**
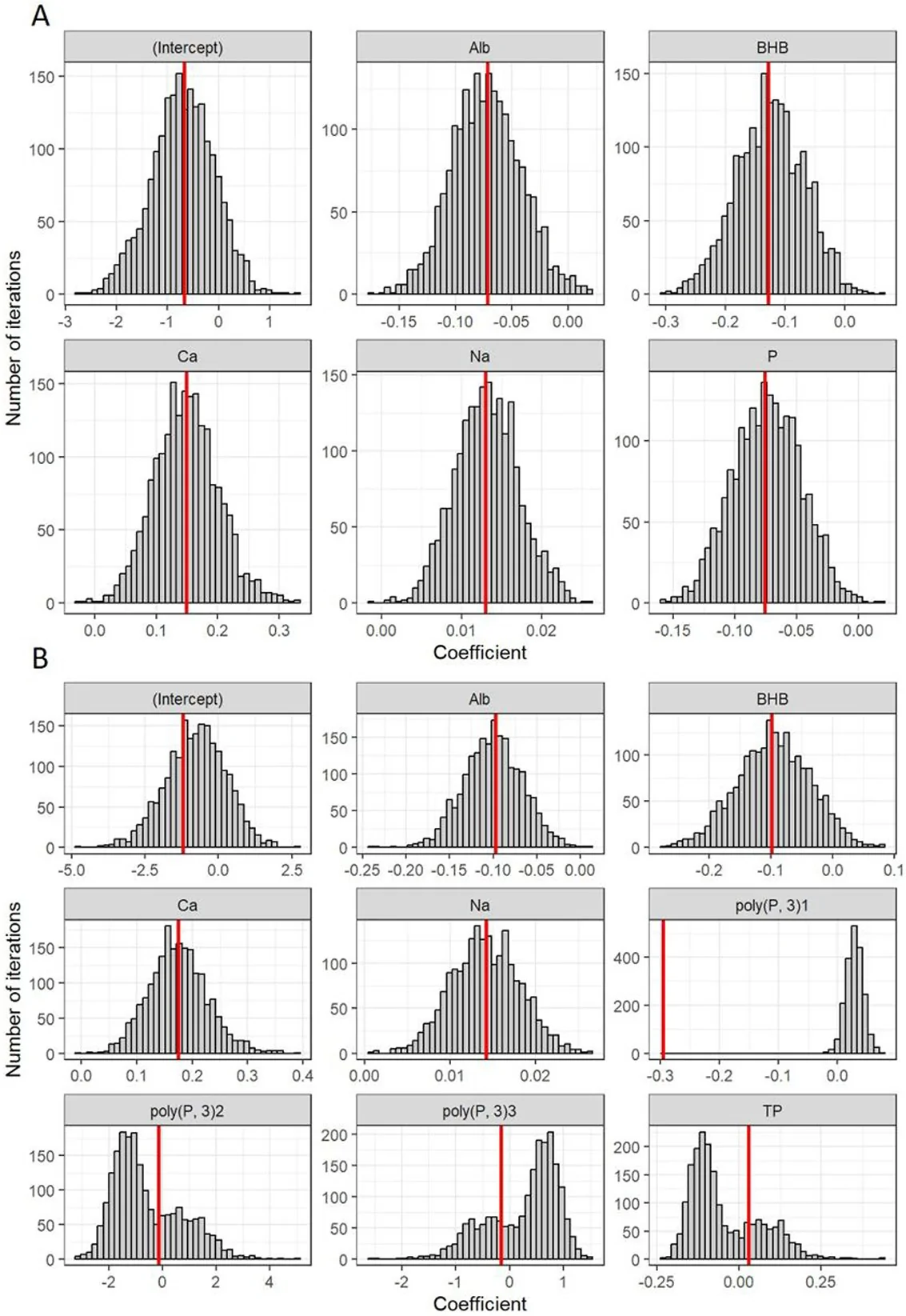
Histograms show the bootstrap distributions of regression coefficients obtained from 2000 resamples for the post-lambing data, including linear (A) and nonlinear (B) models. Each panel corresponds to the intercept and predictor variables retained in the final models. The red vertical line represents the coefficient estimate from the original dataset. The degree of agreement between the original estimate and the bootstrap distributions indicates the stability and robustness of each predictor.

Final model selection was based on graphical inspection of the bootstrap coefficient distributions. After this evaluation, the linear models were retained as the primary models for both pre- and post-lambing datasets. In the linear models, the coefficients estimated from the original dataset were closely aligned with the center of the bootstrap distributions, indicating stable variable estimates across resampled datasets (Figure 3). In contrast, the nonlinear models showed worse agreement between the original coefficients and the bootstrap distributions, particularly for several polynomial terms, suggesting decreased coefficient stability and a higher risk of overfitting (Figure 4). Consequently, the linear models were considered more robust and were selected for the final evaluation based on visual assessment of the residuals.

## Discussion

We evaluated the diagnostic performance of tCa for detecting decreased blood iCa^2+^ and investigated factors associated with iCa^2+^ during the pre- and post-lambing periods in ewes. The results indicated that tCa had moderate diagnostic accuracy for identifying decreased blood iCa^2+^ during the pre-lambing period, whereas its diagnostic performance was limited during the post-lambing period. In addition, multivariable analysis indicated that blood iCa^2+^ during the pre-lambing period was associated with tCa, BHB, pCO₂, and pO₂. In contrast, during the post-lambing period, iCa^2+^ was associated with tCa, phosphorus, Na^+^, total protein, albumin, and BHB.

Unlike cattle, ewes experience challenges in maintaining calcium homeostasis from several weeks before parturition to the early weeks of lactation, driven by increased calcium demands associated with fetal mineralization and the onset of lactation.^19^ The magnitude of calcium demand and the corresponding physiological adaptations play key roles in determining whether hypocalcemia develops, and are influenced by parity and production stage. Primiparous ewes are more prone to hypocalcemia during the prepartum period, whereas multiparous ewes tend to exhibit lower calcium status during lactation.^20^ In agreement with this report, multiparous Assaf ewes in our study exhibited lower blood iCa^2+^ during the post-lambing period compared with the pre-lambing period. In sheep, physiological adaptation to increased calcium demand primarily relies on the mobilization of calcium from bone reserves rather than increased intestinal absorption.^21^ Therefore, the ability to effectively mobilize bone calcium becomes critical for maintaining calcium homeostasis. In older, multiparous ewes, decreased ability to mobilize calcium from bone may limit this adaptive response.^22^ Furthermore, in high-yielding dairy breeds such as Assaf sheep, increased calcium loss associated with early lactation, combined with the high calcium content of sheep milk,^19^ may further exacerbate this imbalance, contributing to the observed decrease in iCa^2+^ during the post-lambing period.

Several studies have compared calcium status between the pre- and post-lambing periods in ewes, primarily based on tCa. Overall, these studies generally report lower tCa during late gestation and an increase during the early lactation period. One study reported that serum tCa was significantly lower in dry pregnant Chios ewes as compared with lactating non-pregnant ewes, regardless of parity.^23^ Similarly, in Awassi ewes, tCa was lower during late gestation compared with postpartum days 7 and 14.^24^ In Lacaune and German Blackhead mutton ewes, the lowest tCa was observed around parturition, particularly on the first day of lactation, followed by a steady increase during lactation.^25^^,^^26^ Collectively, these findings suggest that tCa tends to increase during the post-lambing period compared with the pre-lambing period, although some variation exists among breeds. Consistent with these reports, tCa in our study also was lower during the pre-lambing period. However, when iCa^2+^ was considered, a contrasting pattern emerged, with lower concentrations observed during the post-lambing period. This discrepancy indicates that tCa may not fully reflect iCa^2+^ status, particularly during periods of physiological transition.

The agreement between iCa^2+^ and tCa has not been extensively investigated in ewes, with only a limited number of studies addressing this topic. An earlier study reported that although tCa and iCa^2+^ were correlated in ewes, the correlation was not sufficiently strong to allow reliable prediction of iCa^2+^ from tCa.^27^ Similarly, another study investigated the utility of tCa and iCa^2+^ for assessing calcium status in dry Chios ewes.^28^ Notably, none of the ewes were classified as hypocalcemic based on tCa, whereas 25 out of 30 ewes were identified as hypocalcemic when assessment was based on iCa^2+^. However, previous studies have not evaluated the diagnostic performance of tCa for predicting the true calcium status in ewes. This evaluation is clinically relevant, because serum tCa remains widely used in practice to assess calcium status, despite evidence from both sheep and cattle studies indicating that tCa does not accurately reflect true calcium status.^10^^,^^11^^,^^27^ In our study, the diagnostic performance of tCa for detecting decreased blood iCa^2+^ varied markedly between the pre- and post-lambing periods. During the pre-lambing period, tCa demonstrated moderate discriminating ability for detecting decreased iCa^2+^ with an AUC of 0.73. In contrast, its discriminating ability during the post-lambing period was poor, with an AUC of 0.59. This value indicates that tCa distinguished hypocalcemic from normocalcemic ewes only marginally better than chance during the post-lambing period. In practical terms, when a hypocalcemic ewe (based on iCa^2+^) is compared with a normocalcemic ewe, the probability that the hypocalcemic ewe will have a lower tCa is only 59%. Consequently, in 41% of cases, tCa would paradoxically be higher in hypocalcemic animals than in normocalcemic ones. These results strongly suggest that tCa is an unreliable indicator for diagnosing hypocalcemia in Assaf ewes, especially during post-lambing period.

Albumin possesses a net negative charge at physiological pH, enabling it to bind certain cations and thereby serve as a buffer for iCa^2+^.^6^^,^^29^ Its calcium-binding capacity is strongly pH-dependent, such that decreases in blood pH decrease binding affinity and increase iCa^2+^, whereas alkalinization enhances binding and lowers iCa^2+^. In our study, neither albumin nor blood pH was associated with iCa^2+^ during the pre-lambing period, a finding consistent with previous observations in ewes showing no relationship between iCa^2+^, blood pH, and plasma protein concentrations during early pregnancy.^27^ In contrast, a significant inverse association between albumin and iCa^2+^ was identified during the postpartum period, independent of blood pH.^29^ This divergence between physiological stages may reflect alterations in albumin–iCa^2+^ interactions occurring around parturition, although the underlying mechanisms remain unclear. Notably, blood pH was tightly regulated within a narrow range in both periods, which may have limited ability to detect pH-driven variation in iCa^2+^. Given the well-established pH dependency of albumin–calcium binding, this restricted variability in pH likely attenuated any observable influence of pH on iCa^2+^.^30^^,^^31^ Support for pH-independent regulation of iCa^2+^ in ewes also is provided by nutritional studies.^32^^,^^33^ Although in dairy cattle anionic diets commonly are used to induce compensatory metabolic acidosis and increase circulating iCa^2+^,^31^^,^^34^ similar dietary manipulation in ewes does not consistently result in measurable changes in blood or urine pH.^32^^,^^33^ Nevertheless, increased iCa^2+^ concentrations have been reported in ewes fed low dietary cation-anion difference diets, suggesting that mechanisms other than systemic acid–base alterations contribute to calcium regulation in this species.^33^ Taken together, these findings indicate that, under physiological conditions in ewes, the relationship between albumin and iCa^2+^ may be less dependent on blood pH than is typically described in cattle, and may instead be influenced by stage-specific regulatory mechanisms operating during the periparturient period.

During the pre-lambing period, a weak positive association between pCO₂ and blood iCa^2+^ was observed. This relationship is consistent with finding reported in cattle.^11^ These results suggest that a respiratory-origin acidotic state may contribute to increased blood iCa^2+^ during this stage. In addition, an unexpected positive association between blood pO₂ and iCa^2+^ was detected. Although blood samples were collected with careful attention to anaerobic conditions, this finding may be explained by the post-sampling permeability of plastic syringes to oxygen.^35^

In ewes, calcium and BHB are closely linked during the periparturient period.^22^ Previous studies have focused predominantly on calcium status in ewes experiencing negative energy balance, consistently reporting decreased calcium concentrations in hyperketonemic animals.^36^^,^^37^ In addition, decreased calcium status may contribute to increased BHB concentrations in ewes, implying that hypocalcemia may precede or promote ketogenesis.^5^ However, the potential effect of BHB on iCa^2+^ concentrations has remained largely unexplored in this species. In our study, serum BHB was inversely associated with blood iCa^2+^, with this relationship being more pronounced during the post-lambing period. This finding is consistent with previous observations suggesting that increased BHB concentrations may decrease the fraction of ionized calcium within total calcium.^9^ One possible explanation for this association is that calcium distribution in the circulation can be influenced by increases in organic and inorganic anions, which form complexes with ionized calcium, including hydroxybutyrate.^35^

In our study, an inverse relationship between phosphorus and iCa^2+^ was identified during the post-lambing period in ewes. This finding is consistent with earlier observations in sheep demonstrating a reciprocal relationship between circulating phosphate and calcium concentrations, whereby increases in plasma phosphate are associated with decreases in blood calcium concentrations.^38^^,^^39^ Several mechanisms may explain this inverse association. At the physicochemical level, phosphate can directly bind to iCa^2+^ in the circulation, thereby decreasing the biologically active calcium fraction.^40^ This interaction has been shown to occur across physiological pH ranges and represents a direct effect of phosphate on calcium partitioning in blood.^41^ In addition, the relationship between phosphorus and calcium in sheep is strongly influenced by bone metabolism. Unlike monogastric species, calcium homeostasis in sheep relies predominantly on bone mobilization rather than intestinal absorption.^42^ During periods of increased calcium demand, such as the periparturient period, bone resorption leads to the simultaneous release of both calcium and phosphate into the circulation.^21^ If the released phosphate is not efficiently cleared, circulating phosphate concentrations may increase and subsequently decrease iCa^2+^ through complex formation, reinforcing the inverse relationship observed in our study.^21^ Furthermore, the limited capacity for renal phosphate excretion in sheep may exacerbate this interaction. In contrast to monogastric species, where phosphaturia plays a key regulatory role, sheep exhibit minimal urinary phosphate excretion, allowing circulating phosphate to accumulate more readily.^43^ This may further enhance the suppressive effect of phosphate on iCa^2+^, particularly under conditions of active bone turnover.

A weak positive association between serum Na^+^ concentration and blood iCa^2+^ was observed during the post-lambing period. This relationship is unlikely to be explained by direct competition between Na^+^ and iCa^2+^ for protein-binding sites. A previous study demonstrated that, at physiological pH, albumin exhibits minimal binding affinity for monovalent cations such as Na^+^ and K^+^, unlike its measurable interactions with certain anions such as chloride (Cl^-^), whereas divalent cations, including iCa^2+^, are substantially bound.^44^ This study indicates that Na^+^ remains largely free in solution and does not directly displace calcium from albumin binding sites. A more plausible explanation is provided by changes in ionic strength. Another previous study showed that increasing ionic strength through NaCl addition enhanced the dissociation of calcium from serum proteins, thereby increasing iCa^2+^, particularly in whole serum.^45^ Accordingly, an increase in circulating Na^+^ would increase the overall ionic strength of the blood, weakening electrostatic interactions between iCa^2+^ and negatively-charged binding sites on serum proteins and promoting a shift toward the ionized form.

Our study had some limitations. The findings are based on a single flock of a single breed (Assaf), which may limit their generalizability to other sheep breeds or management systems. The study employed a 2 time-point cross-sectional design rather than serial sampling of the same animals through parturition, which prevents a detailed analysis of the temporal dynamics immediately surrounding lambing. Furthermore, the sampling intervals were variable among animals, and key hormonal regulators of calcium metabolism, such as parathyroid hormone and vitamin D, were not measured. Another limitation relates to the modeling approach. External validation of the developed models could not be performed using an independent dataset. Although internal validation was conducted using bootstrap resampling, this approach relies on repeated sampling from the same dataset (*n* = 105) and may therefore not fully capture model performance in external populations. Finally, the dietary cation-anion difference was not assessed, precluding a direct analysis of its impact on the observed acid–base changes.

## Conclusion

Our findings demonstrate that blood iCa^2+^ varies in a production stage–dependent manner in Assaf ewes. The results support the initial hypothesis of the study that the association between tCa and iCa^2+^ differs between the pre- and post-lambing periods, and that tCa does not consistently reflect iCa^2+^ status across these stages. Although tCa showed moderate diagnostic utility during the pre-lambing period, its poor performance in the post-lambing period highlights a substantial diagnostic discordance with biologically active iCa^2+^. These findings support the clinical relevance of direct measurement of iCa^2+^ for the accurate assessment of hypocalcemia in Assaf ewes, particularly during the post-lambing period. The observed relationship between iCa^2+^ and selected biochemical, acid–base, mineral, and metabolic variables partially supports the second hypothesis of the study that measured iCa^2+^ during these periods is influenced by changes in acid–base status, plasma protein concentrations, mineral balance, and metabolic indicators. However, the limited explanatory power of the multiple linear regression models for blood iCa^2+^ changes may, in part, reflect the absence of key regulatory factors, such as hormonal mediators of calcium homeostasis, which were not assessed in this clinically healthy population. Future studies incorporating longitudinal designs and comprehensive evaluation of hormonal and metabolic regulators are warranted to better elucidate the mechanisms underlying these stage-dependent differences and to refine diagnostic approaches for hypocalcemia in ewes.

## Supplementary Material

Supplementary_Table_1_aalag202

Supplementary_Table_2_aalag202

Supplementary_Table_3_aalag202

## Off-label antimicrobial declaration

Authors declare no off-label use of antimicrobials.

## Institutional animal care and use committee or other approval declaration

Approved by the Kastamonu University Animal Experiments Local Ethics Committee (23/05/2025, Decision No: 39).

## Appendix

### Synchronization protocol and feeding of the ewes

The ewes were synchronized using intravaginal sponges containing 20 mg of fluorogestone acetate (Chronogest, MSD Animal Health, Turkey) for a period of 7 days. On the day of sponge removal, each ewe was given 350 IU of equine chorionic gonadotropin (Folligon, MSD Animal Health). All ewes were allowed to mate naturally. To determine the mating date, rams were introduced into the flock at the time of sponge removal. The day on which mating was observed was recorded as the day of mating (day 0). The feeding regimen of the flock was determined by the farm owner in accordance with recommendations provided by the attending private veterinarian. The composition of the rations based on the forage-to-concentrate ratio was 49.5% forage and 50.5% concentrate during the close-up period, and 46% forage and 54% concentrate during the lactation period. The feed materials used in the rations were as follows: straw, alfalfa, a grain mixture (Hungarian vetch, feed pea, wheat, and triticale), barley silage, corn silage, barley, soybean meal, bypass fat, buffer, and a commercial dairy compound feed.

### Blood sampling and laboratory analyses

A 2-stage sampling protocol was implemented. During the first farm visit, blood samples were collected from all pregnant ewes (*n* = 105) on the same day during late gestation. The precise gestational stage at the time of sampling was determined retrospectively by recording and monitoring individual lambing dates for each ewe. After completion of all lambings within the flock, a second farm visit was conducted. Blood samples were collected again from the same animals during early lactation. The day in milk (DIM) at the time of the second sampling was calculated individually for each ewe based on the recorded date of parturition. In each sampling period, blood was collected from the jugular vein of each ewe. Samples were obtained under anaerobic conditions into syringes containing dried heparin for blood gas analysis, K₃EDTA tubes for PCV determination, and lithium heparin tubes for plasma biochemical analyses. The variables obtained from the blood gas and electrolyte analyses and included in the statistical analyses were blood pH, partial pressure of carbon dioxide (pCO₂), partial pressure of oxygen (pO₂), sodium (Na^+^), potassium (K^+^), iCa^2+^, total carbon dioxide (TCO₂), and bicarbonate (HCO₃^−^). The concentrations of blood Na^+^, K^+^, and iCa^2+^ were determined using ion-selective electrode potentiometry; blood pH and pCO₂ were measured by direct potentiometric method, and blood pO₂ was measured using an amperometric method.

Blood samples collected into lithium heparin tubes were centrifuged immediately after collection and stored at −20 °C for a maximum of one month until biochemical analysis. The measured biochemical variables included alkaline phosphatase (AP-kinetic photometric method), tCa (Phosphonazo III method), total protein (biuret method), albumin (bromocresol green method), glucose (glucose oxidase method), β-hydroxybutyrate (BHB, enzymatic determination using β-hydroxybutyrate dehydrogenase), phosphorus (ammonium molybdate method), and magnesium (xylidyl blue method). The analyzer was calibrated and quality-controlled using manufacturer-provided reference solutions at the beginning of each measurement day. Calibration and quality control procedures were repeated whenever a reagent kit was replaced during the same day. Control measurements obtained across the 5 measurement days were used to calculate the inter-assay coefficient of variation (CV). Intra-assay CV was determined by performing 5 replicate measurements for each variable within the same measurement day.

### Statistical analysis

Three stages of statistical analysis were performed. First, differences in continuous variables between the pre- and post-lambing periods were assessed using the Wilcoxon rank-sum test. Second, optimal cut-off values of tCa for the identification of hypocalcemia were determined separately for the pre- and post-lambing periods using *cutpointr* package in R.^17^ Hypocalcemia in both periods was defined as a blood iCa^2+^ concentration < 1.1 mmol/L.^18^ Receiver operating characteristic (ROC) curves were constructed for both periods, and the area under the receiver operating characteristics curve (AUC) was used to quantify discriminatory performance. The AUC values were interpreted as follows: highly accurate (0.9 < AUC < 1.0), moderately accurate (0.7 < AUC ≤ 0.9), slightly accurate (0.5 < AUC ≤ 0.7), and inaccurate (AUC ≤ 0.5).^46^ Internal validation and variability evaluation of the estimated tCa cut-off values were performed using a bootstrap resampling. For each period, 1000 bootstrap samples with replacement were generated by resampling with replacement from the original dataset, and the optimal cut-off was re-estimated separately in the in-bag and out-of-bag samples. To determine whether the bootstrap-derived cut-offs were practically equivalent to the estimated optimal cut-off for each period, a region of practical equivalence (ROPE) was defined as ±5% of the corresponding optimal cut-off value. This range can be considered as threshold values, which are very similar from a clinical application perspective.^47^ The proportion of bootstrap cut-points falling within this interval was calculated. In addition, performance metrics, including the AUC, sensitivity, specificity as well as overall accuracy, were calculated for both in-bag and out-of-bag samples and compared with those obtained from the optimal cut-off.

Finally, the associations between blood iCa^2+^ and both measured and calculated variables including blood pH, pCO₂, pO₂, Na^+^, K^+^, TCO₂, HCO₃^−^, AP, tCa, phosphorus, magnesium, total protein, albumin, glucose, and BHB were evaluated separately for the pre- and post-lambing periods. In both periods, fetal number also was included as a predictor. Additionally, the interval between blood sampling and lambing was evaluated in the pre-lambing period, whereas the time from lambing to sampling was evaluated in the post-lambing period. Two complementary modeling approaches were applied. In the first approach, a potential nonlinear relationship was explored for each continuous predictor by comparing linear, quadratic and cubic transformations. Subsequently, for each predictor, the transformation yielding the lowest Akaike information criterion (AIC) values in univariable analysis was selected for inclusion in further multivariable modeling. In the second approach, all predictors were modeled assuming linear relationship to obtain more robust estimates and to minimize the potential influence of outliers. For both approaches, selected variables were entered into the multivariable models, and a manual backward stepwise selection procedure was applied. At each step, the independent variable with the highest non-significant p-value (*P* ≥ .05) was sequentially removed until all remaining variables were statistically significant (*P* < .05). Internal validation of the multivariable models obtained from each approach was performed using bootstrap resampling to evaluate coefficient stability and potential overfitting. A total of 2000 bootstrap samples were generated by resampling with replacement from the original dataset, and the final model was refitted in each bootstrap sample. The distribution of regression coefficients obtained from bootstrap sample were examined graphically using histograms. The coefficients estimated from the original dataset were overlaid on the bootstrap distributions to evaluate their agreement with the resampled estimates. Close alignment between the original coefficients and the center of bootstrap distributions was interpreted as evidence of stable variable estimates and limited overfitting.

## Abbreviations

AIC: Akaike information criterion
AP: alkaline phosphatase
AUC: area under the receiver operating characteristic curve
BHB: β-hydroxybutyrate
CI: confidence interval
CV: coefficient of variation
DIM: days in milk
HCO₃^-^: bicarbonate
IQR: interquartile range
iCa^2+^: ionized calcium concentration
K^+^: potassium
Na^+^: sodium
pCO₂: partial carbon dioxide pressure
PCV: packed cell volume
pO₂: partial oxygen pressure
ROC: receiver operating characteristic
ROPE: region of practical equivalence
tCa: total calcium concentration
TCO₂: total carbon dioxide
