# Supplementary material for: Discriminatory performance of total calcium for ionized hypocalcemia and factors affecting ionized calcium in Assaf ewes during the pre- and post-lambing periods
Source: J Vet Intern Med. 2026 Sep 7;40(5):aalag202. doi: 10.1093/jvimsj/aalag202 (PMC13549433; doi:10.1093/jvimsj/aalag202)
Supplement: Supplementary file 1 [file supplementary_table_1_aalag202.docx]

**Supplementary Table 3.** Linear and nonlinear multivariable analysis of the association between blood iCa²⁺ and selected biochemical variables in the postlambing dataset (n = 105)

|  | iCa²⁺ | | |  | iCa²⁺ | | |
| --- | --- | --- | --- | --- | --- | --- | --- |
| *Predictors* | *Estimates* | *CI* | *p* |  | *Estimates* | *CI* | *p* |
| (Intercept) | -0.67 | -1.85 – 0.51 | 0.265 |  | -1.20 | -2.42 – 0.01 | 0.052 |
| Alb | -0.07 | -0.13 – -0.01 | **0.028** |  | -0.10 | -0.16 – -0.03 | **0.003** |
| BHB | -0.13 | -0.22 – -0.04 | **0.006** |  | -0.10 | -0.19 – -0.01 | **0.037** |
| Ca | 0.15 | 0.04 – 0.25 | **0.006** |  | 0.17 | 0.07 – 0.28 | **0.001** |
| Na | 0.01 | 0.01 – 0.02 | **0.001** |  | 0.01 | 0.01 – 0.02 | **<0.001** |
| P | -0.08 | -0.11 – -0.04 | **<0.001** |  |  |  |  |
| P [1st degree] |  |  |  |  | -0.30 | -0.45 – -0.15 | **<0.001** |
| P [2nd degree] |  |  |  |  | -0.14 | -0.29 – 0.01 | 0.063 |
| P [3rd degree] |  |  |  |  | -0.15 | -0.30 – -0.00 | **0.049** |
| TP |  |  |  |  | 0.03 | 0.00 – 0.06 | **0.046** |
| Observations | 105 | | |  | 105 | | |
| R^2^ / R^2^ adjusted | 0.283 / 0.247 | | |  | 0.361 / 0.307 | | |
