# Supplementary material for: Discriminatory performance of total calcium for ionized hypocalcemia and factors affecting ionized calcium in Assaf ewes during the pre- and post-lambing periods
Source: J Vet Intern Med. 2026 Sep 7;40(5):aalag202. doi: 10.1093/jvimsj/aalag202 (PMC13549433; doi:10.1093/jvimsj/aalag202)
Supplement: Supplementary file 2 [file supplementary_table_2_aalag202.docx]

**Supplementary Table 2.** Linear and nonlinear multivariable regression models of the association between blood iCa²⁺ and selected biochemical variables in the prelambing dataset (n = 105)

|  | iCa²⁺ | | | iCa²⁺ | | | | iCa²⁺ | | | | iCa²⁺ | | | |
| --- | --- | --- | --- | --- | --- | --- | --- | --- | --- | --- | --- | --- | --- | --- | --- |
| *Predictors* | *Estimates* | *CI* | *P-value* | | *Estimates* | *CI* | *P-value* | | *Estimates* | *CI* | *P-value* | | *Estimates* | *CI* | *P-value* |
| (Intercept) | 0.22 | -0.13 – 0.58 | .21 | | 0.63 | 0.39 – 0.87 | **<.001** | | 0.34 | 0.02 – 0.67 | **.04** | | 0.82 | 0.69 – 0.96 | **<.001** |
| Alb | 0.05 | -0.01 – 0.11 | .1 | | 0.06 | -0.00 – 0.11 | .06 | |  |  |  | |  |  |  |
| BHB | -0.02 | -0.04 – -0.00 | **.03** | |  |  |  | | -0.03 | -0.05 – -0.00 | **.02** | |  |  |  |
| Ca | 0.18 | 0.07 – 0.30 | **.002** | |  |  |  | | 0.21 | 0.10 – 0.32 | **<.001** | |  |  |  |
| PCO2 | 0.00 | 0.00 – 0.00 | **<.001** | | 0.00 | 0.00 – 0.00 | **<.001** | | 0.00 | 0.00 – 0.00 | **<.001** | | 0.00 | 0.00 – 0.00 | **<.001** |
| PO2 | 0.01 | 0.00 – 0.01 | **<.001** | | 0.01 | 0.00 – 0.01 | **<.001** | | 0.01 | 0.00 – 0.01 | **<.001** | | 0.01 | 0.00 – 0.01 | **<.001** |
| BHB [1st degree] |  |  |  | | -0.14 | -0.26 – -0.02 | **.03** | |  |  |  | | -0.15 | -0.28 – -0.02 | **.02** |
| BHB [2nd degree] |  |  |  | | 0.01 | -0.11 – 0.14 | .82 | |  |  |  | | -0.00 | -0.13 – 0.12 | .98 |
| BHB [3rd degree] |  |  |  | | -0.21 | -0.33 – -0.09 | **.001** | |  |  |  | | -0.21 | -0.33 – -0.09 | **.001** |
| Ca [1st degree] |  |  |  | | 0.23 | 0.10 – 0.37 | **.001** | |  |  |  | | 0.27 | 0.14 – 0.40 | **<.001** |
| Ca [2nd degree] |  |  |  | | -0.20 | -0.32 – -0.08 | **.002** | |  |  |  | | -0.19 | -0.31 – -0.07 | **.002** |
| Observations | 105 | | | 105 | | | | 105 | | | | 105 | | | |
| R^2^ / R^2^ adjusted | 0.302 / 0.267 | | | 0.433 / 0.385 | | | | 0.283 / 0.254 | | | | 0.411 / 0.369 | | | |
